# A retrospective multicentre clinical study on management of isolated splenic vein thrombosis: risks and benefits of anticoagulation

**DOI:** 10.1007/s00423-024-03295-y

**Published:** 2024-04-09

**Authors:** A. M. Eltweri, M. Basamh, Y. Y. Ting, M. Harris, G. Garcea, L. L. Kuan

**Affiliations:** 1 Hepatobiliary and Pancreatic Surgery Department, University Hospitals of Leicester, Leicester General Hospital, Leicester, UK; 2 Department of Surgery, The Queen Elizabeth Hospital, The University of Adelaide, Adelaide, South Australia Australia

**Keywords:** Isolated, Splenic vein, Thrombosis, Management, Anticoagulation

## Abstract

**Introduction:**

Isolated splenic vein thrombosis (iSVT) is a common complication of pancreatic disease. Whilst patients remain asymptomatic, there is a risk of sinistral portal hypertension and subsequent bleeding from gastric varices if recanalisation does not occur. There is wide variation of iSVT treatment, even within single centres. We report outcomes of iSVT from tertiary referral hepatobiliary and pancreatic (HPB) units including the impact of anticoagulation on recanalisation rates and subsequent variceal bleeding risk.

**Methods:**

A retrospective cohort study including all patients diagnosed with iSVT on contrast-enhanced CT scan abdomen and pelvis between 2011 and 2019 from two institutions. Patients with both SVT and portal vein thrombosis at diagnosis and isolated splenic vein thrombosis secondary to malignancy were excluded. The outcomes of anticoagulation, recanalisation rates, risk of bleeding and progression to portal vein thrombosis were examined using CT scan abdomen and pelvis with contrast.

**Results:**

Ninety-eight patients with iSVT were included, of which 39 patients received anticoagulation (40%). The most common cause of iSVT was acute pancreatitis *n* = 88 (90%). The recanalisation rate in the anticoagulation group was 46% vs 15% in patients receiving no anticoagulation (*p* = *0.0008*, OR = 4.7, 95% CI 1.775 to 11.72). Upper abdominal vascular collaterals (demonstrated on CT scan angiography) were significantly less amongst patients who received anticoagulation treatment (*p* = *0.03*, OR = 0.4, 95% CI 0.1736 to 0.9288). The overall rate of upper GI variceal-related bleeding was 3% (*n* = 3/98) and it was independent of anticoagulation treatment. Two of the patients received therapeutic anticoagulation.

**Conclusion:**

The current data supports that therapeutic anticoagulation is associated with a statistically significant increase in recanalisation rates of the splenic vein, with a subsequent reduction in radiological left-sided portal hypertension. However, all patients had a very low risk of variceal bleeding regardless of anticoagulation. The findings from this retrospective study should merit further investigation in large-scale randomised clinical trials.

## Introduction

Isolated splenic vein thrombosis (iSVT) was first reported during post-mortem examination about 100 years ago by Frick A [1]. It represents a small number of splanchnic vein thromboses and the most common cause is acute or chronic pancreatitis [2]. The symptomatic splenic vein thrombosis was defined as those patients having signs and symptoms of gastrointestinal (GI) bleeding along with the symptoms of chronic pancreatitis and those who do not have these symptoms are considered asymptomatic [3]. The incidence of iSVT amongst patients with pancreatitis has been reported between 2.7 and 20% [4–8]. Butler JR et al. reported pancreatitis-induced SVT in a meta-analysis as 22.6% in patients with acute pancreatitis and 12.4% in patients with chronic pancreatitis [9]. The incidence of iSVT is difficult to predict [6] and it is likely to be higher than that previously reported, as the majority of patients are without overt symptoms [6, 10] and the increased routine use of cross-sectional imaging will identify more patients. However, symptomatic patients usually have abdominal pain as a common presenting symptom, and if GI varices develop, they might present with GI bleeding [6].

The management of common sites venous thrombosis, such as deep vein thrombosis or pulmonary embolisms, is with anticoagulation for at least 3 months if provoked and 6 months or longer if it is unprovoked [11]. Prolonged anticoagulation treatment is not applicable to most patients with splanchnic vein thrombosis due to the risk of bleeding [12]. The recommended management of patients with upper GI bleeding secondary to splenic vein thrombosis is splenectomy [10, 13].

Whether short-term (circa 3 to 6 months) anticoagulation for acute iSVT is warranted to improve recanalisation and reduce the risk of development of oesophagogastric varices is unknown. To our knowledge, this retrospective clinical study contains the largest clinical series of patients presenting with iSVT with subsequent outcomes after anticoagulation.

## Methods

The use of anonymised patient information was approved by the institutional clinical and effectiveness board. Individual patient consent was not required for the purpose of this observational clinical study.

### Study population

Patients with a confirmed radiological diagnosis of iSVT at the University Hospitals of Leicester NHS Trust (UK) and Central Adelaide Local Health Network (The Royal Adelaide and the Queen Elizabeth Hospitals) (Australia) between January 2011 and December 2019 were reviewed. Patients were excluded if concomitant portal vein and splenic vein thromboses were identified at diagnosis or if the underlying cause was malignancy. The diagnosis of iSVT was made by abdominal contrast-enhanced computed tomography (CT) scan.

### Data collection

The data collection and presentation of this retrospective clinical study was conducted according to the strengthening the reporting of observational studies guidelines. Data were collected from a prospectively maintained electronic system that contained a complete record of each patient’s hospitalisation including discharge letters, medications and subsequent procedures (radiological and/or endoscopic). The following data were collected: progression to portal vein thrombosis, aetiology of thrombosis, radiological evidence of intra-abdominal collaterals and endoscopic oesophagogastric varices, treatment provided, haemorrhagic events and mortality in both groups.

### Statistical analysis

The demographic data is presented as percentage and number out of the total. The differences between the two groups were tested for statistical significance using Pearson chi-square test for all categorical data, *p* value, odd ratio and 95% confidence interval were presented.

## Results

### Patient demographics

A total of 165 patients were identified to have splenic vein thrombosis. Sixty-seven patients were excluded (35 had a malignancy as an underlying cause and 32 had combined splenic/portal vein thrombosis at presentation). Of the 98 included patients with iSVT, 59 patients did not receive anticoagulation. The most common cause of iSVT in this cohort was acute pancreatitis *n* = 88, (90%) (Table 1).

**Table 1:** Patients’ demographics in the anticoagulation vs no anticoagulation group

Variables	Anticoagulation (*n* = 39)	No anticoagulation (*n* = 59)	*p* value
Male *n* (%)	25 (64%)	42 (71%)	> 0.999
Age (mean)	53 years	53.5 years	
Causes of iSVT *n* (%)			
• Severe acute pancreatitis	20 (51%)	34 (58%)	> 0.999
• Acute pancreatitis	12 (31%)	22 (37%)	> 0.999
• Sepsis	2 (5%)	2 (3%)	> 0.999
• Coagulation disorders	5 (13%)	1 (2%)	= 0.0707

Severe acute pancreatitis and acute pancreatitis were defined radiologically on CT scan abdomen and pelvis

### Anticoagulation treatment

Thirty-nine patients received anticoagulation treatment for splenic vein thrombosis. Due to the lack of established guidelines regarding the management of iSVT, the anticoagulation treatments and duration were variable and included heparin, low molecular weight heparin (LMWH), warfarin or novel oral anticoagulants (Table 2). The decision to anti-coagulate and type of anti-coagulant was predominantly down to an individual consultant’s practice regarding management of iSVT and not based on any standard operating policy.

**Table 2:** Types and duration of anticoagulation therapy

	1–6 months	7–12 months	> 12 months	Not specified
Heparin + warfarin or NOAC	*-*	*-*	*n* = *1*	*n* = *1*
LMWH + warfarin	*n* = *10*	*n* = *1*	*n* = *2*	*n* = *2*
LMWH + NOAC	*n* = *2*	*-*	*n* = *2*	
LMWH	*n* = *6*	*-*	*-*	*n* = *6*
Warfarin only	*n* = *1*	*-*	*n* = *2*	*-*
NOAC only	*n* = *1*	*-*	*n* = *2*	*-*

*NOAC*, novel oral anticoagulant; *LMWH*, low molecular weight heparin

*n*, number of patients

### Recanalisation with or without anticoagulation therapy

The recanalisation rate was statistically greater in the anticoagulation group, and this was seen in 18 patients (46%) (*p* = 0.0008, OR = 4.7, 95% CI 1.775 to 11.72). Amongst the patients who did not receive anticoagulation, nine patients (15%) had recanalisation of the splenic vein. The development of perigastric vascular collaterals, evident on CT scan angiography, was significantly less amongst patients who received anticoagulation (*p* = 0.03, OR = 0.4, 95% CI 0.1736 to 0.9288). The progression of thrombosis to portal vein was seen in both groups but statistically not affected by anticoagulation (*p* = 0.1495, OR 3.39, 95% CI 0.895 to 12.87). Table 3 summarises the outcomes in each group.

**Table 3:** Outcomes with anticoagulation vs without anticoagulation

	With anticoagulation *n* = 39	Without anticoagulation *n* = 58	*p* value
Recanalisation of splenic vein	18 (46%)	9 (15%)	*p* = 0.0008
Oesophagogastric (OG) varices	2 (5%)	5 (9%)	*p* > 0.9999
Variceal bleeding	2 (5%)	1 (2%)	*p* = 0.6950
Inpatient mortality	1 (3%)	1 (2%)	*p* = 0.9739

### Development of oesophagogastric (OG) varices, variceal bleeding and mortality

The identification of oesophagogastric varices endoscopically in both groups was seen in two patients (5%) who received anticoagulation and five patients (8%) did not receive anticoagulation, and the difference was not statistically significant. The rate of long-term upper GI variceal bleeding amongst patients treated with or without anticoagulation was *n* = 2 (5%) vs *n* = 1 (2%) respectively and again not statistically significant. Three patients required endoscopic treatment, in the form of adrenaline injection, cyanoacrylate injection and hystacryl glue in lipiodol injection. No patients required a splenectomy. The inpatient mortality rate was one patient in each group secondary to multi-organ failure with no bleeding-related mortality. Table 3 summarises the outcomes in each group.

## Discussion

The principle findings we observed in this retrospective clinical study was that treating iSVT patients with therapeutic dose anticoagulation from the onset of diagnosis, for an average of 5 months, was associated with significantly greater rates of recanalisation of the splenic vein and a reduction in radiologically evident sinistral portal hypertension.

Amitrano L et al. reported a 75% recanalisation rate in anti-coagulated patients with splanchnic vein thromboses amongst cirrhotic patients for 6 months [14]. However, their study population were heterogeneous and anticoagulation treatment was only started after an episode of bleeding from oesophagogastric varices [14]. Others have reported 45.4% recanalisation rates in non-cirrhotic patients with splanchnic vein thromboses received anti-coagulant and lifelong therapy preventing recurrent thrombosis [15]. Plessier et al. reported a 54% recanalisation of splenic vein thrombosis in a subset of splanchnic vein thrombosis patients [16]. The latter study reported that the presence of ascites and splenic vein thrombosis together with portal vein thrombosis carried a poorer prognosis and a reduced probability of recanalisation of either with anticoagulation [16]. These results are broadly in line with our result of recanalisation rates of approximately 46% amongst patients who received anticoagulation treatment. Interestingly, the mortality rate amongst patients with liver cirrhosis and splanchnic vein thrombosis was higher amongst those who did not receive anticoagulation (*p* = 0.006) and similar for re-bleeding from oesophageal varices but it did not reach statistical significance [14].

Unresolved splenic vein thrombosis can lead to the development of collateral vessels to drain blood around the spleen and stomach, via the most common pathway through the short gastrics, left phrenic and gastroepiploic vessels [9, 10, 13, 17]. As a result, there will be an increase in the pressure in the submucosal vessels at the stomach fundus [9, 10, 17]. If splenic vein recanalisation does not occur, it can lead to sinistral portal hypertension and eventually oesophagogastric varices [13], and gastric varices (the latter being more common) [7, 18, 19]. The incidence rate of oesophagogastric varices was variable, some authors reported it as low as 8.1–18% [5, 7, 12] and others reported it as high as 35–55% [4, 6, 19, 20].

The differences in these figures can be explained by the small cohort population, the heterogeneity of the causes and the use of different investigative modalities in making a diagnosis of varices. In our reported cohort of patients, the overall proportion of GI varices amongst the two groups was 7%; two patients in the anticoagulation group and five amongst those who did not receive anticoagulation. Interestingly, Bernades et al. found that if GI varices were not present at the time of diagnosis of splenoportal venous obstruction, it did not occur during an average follow-up of 29 months [5]. Weber et al. also reported a lack of variceal progression during follow-up [10], but others contradict this statement [6]. The contradictions in these reports could be due to the fact that the splenic and portal veins have several tributaries to other anatomical sites. Such as the renal vein via adrenal vein, the left gastroepiploic veins can collateralise to the inferior mesenteric vein as previously reported as a cause of lower gastrointestinal bleeding, and to the inferior vena cava via the diaphragmatic and intercostal veins [10, 21]. Moreover, the lack of surveillance programmes and the variable duration of follow-up may have contributed to it.

Isolated splenic vein thrombosis is a common complication in chronic pancreatitis patients and more so amongst those with a pseudocyst formation and history of smoking [7]. Bernades et al. reported 91.4% of splenoportal venous thrombosis was secondary to pancreatitis and pseudocyst [5]. In our cohort of patients, the diagnosis of iSVT was made by contrast-enhanced CT scan of the abdomen and 90% was identified to be secondary to acute pancreatitis. The contrast-enhanced abdominal CT scan is a notable modality for the diagnosis of splenoportal venous thrombosis [22].

Some studies have reported 8% complete resolution of splenic vein thrombosis after resolution of underlying cause, without the use of anticoagulation [7]. Others have reported there was no recanalisation without anticoagulation therapy [9]. Our study has demonstrated that the recanalisation rate was 15% amongst those who did not receive therapeutic anticoagulation treatment, suggesting that spontaneous recanalisation is possible in some patients. A possible explanation for this recanalisation in our cohort group is all patients have received prophylactic dose LMWH based on their weight and renal function during their hospital stay. As per the National Institute for Health and Care Excellence (NICE) guidelines, all surgical admissions warrant prophylactic anticoagulation for the duration of their hospital stay unless otherwise contraindicated [23].

In our cohort, the overall rate of upper GI variceal-related bleeding was 3% and it was independent of anticoagulation treatment. These patients were managed with endoscopic treatment which was sufficient to control the bleeding. The reported risk of bleeding in the literature amongst patients with iSVT is variable and ranged from 4 to 35% [4–6, 8, 12, 19, 20, 24]. The risk of bleeding from GI varices also varies from CT scan identified varices of 5% compared to those identified on endoscopy at 18% [24]. Interestingly, some authors reported higher GI bleeding rate of 50% [6], but this figure was originated from a subset of patients with iSVT with known varices [6]. Overall, these figures were largely generated from cohorts with small number of patients.

In patients with chronic pancreatitis and splenic vein thrombosis, bleeding from non-variceal upper GI sources is as common as variceal bleeding and thus, it is important to investigate for non-variceal bleeding sources in these patients before considering definitive treatment [7, 23]. Loftus et al. compared splenectomy vs no splenectomy amongst patients with confirmed left-sided portal hypertension and found that a splenectomy did not prevent recurrent upper GI bleeding, when other causes for GI bleeding were identified [24].

To date, there is a paucity of evidence to determine anticoagulation treatment decision due to the fact that there are no randomised controlled trials (RCTs) assessing the impact of anticoagulation treatment in patients with iSVT [25]. The treatment of venous thromboembolism is aimed at achieving recanalisation of the thrombosed vein and preventing subsequent complications. The duration of anticoagulation treatment is determined on whether the cause of thrombosis is provoked or unprovoked [26]. The Baveno V Faculty recommends 3 months of anticoagulation therapy for provoked acute extrahepatic portal vein obstruction (EHPVO) and lifelong therapy if a prothrombotic cause was documented [27]. However, their definition of EHPVO does not include isolated splenic vein thrombosis and the anticoagulation treatment for chronic thrombosis is still controversial at present [27]. Thatipelli et al. reported increased risk of bleeding in a splanchnic vein thrombosis cohort of 832 patients; of those included, only 62 patients had splenic vein thrombosis and only 5% received anticoagulation [12]. Therefore, the recommendation from that study was against prolonged anticoagulation treatment [12]. Ageno W et al. provided a guidance statement for the management of splanchnic venous thrombosis (including splenic vein thrombosis) which dictated that anticoagulation treatment should be considered for all patients with symptomatic splanchnic vein thrombosis with no evidence of active bleeding, for a minimum of three months [25, 28]. LMWH is recommended for at least 3 months in the management of splanchnic vein thrombosis [12]. Hanafy et al. reported in a small randomised clinical trial comparing rivaroxaban versus warfarin in the management of non-neoplastic related portal vein thrombosis and found rivaroxaban was associated with better recanalisation rate and less bleeding complications [29]. The usage of novel oral anticoagulants such as rivaroxaban and apixaban in the management of thromboses of atypical locations including splanchnic vein thrombosis is safe and as effective as LMWH [18].

Isolated SVT is most commonly caused by pancreatitis [9]. The most common cause of bleeding in these patients is pseudoaneurysm formation with a bleeding rate of 69.4%, followed by 22% from other causes indirectly related to pancreatitis such as peptic ulcer disease or varices [30].

In our cohort, none of the patients required a splenectomy for isolated splenic vein thrombosis over the 10-year study period. A few studies reported that the definitive treatment for bleeding patients from oesophagogastric varices with left-sided portal hypertension after the initial resuscitative measures is a splenectomy, as it eliminates the vascular collaterals developed secondary to splenic vein thrombosis [10, 13]. However, others reported that splenectomy should be performed only for a confirmed diagnosis with life-threatening haemorrhage [23, 24]. Agarwal AK et al. suggested splenectomy should be added to any planned operative procedure for pancreatic disease, in asymptomatic patients with evidence of left sided portal hypertension [8]. Alternative measures described of iSVT management in clinically unfit patients for splenectomy was splenic artery embolisation [31], but this carries the risk of developing splenic infarct, and may subsequently lead to abscess formation.

This study is the largest clinical cohort of iSVT in the literature comparing the outcomes of anticoagulation in patients with iSVT versus conservative management. It included two international HPB units across two continents. Despite attempts to keep the study group as homogenous as possible, there are limitations. Its retrospective nature is a significant one, limited generalisability and the fact that the choice between anticoagulation or not was largely down to individual clinician preferences is a confounding factor. Furthermore, the duration and type of anticoagulations also varied between patients.

## Conclusion

Our study showed that anticoagulation was associated with a statistically significant increased rate of splenic vein recanalisation and a significant reduction in radiologically evident left-sided portal hypertension. Anticoagulation appeared safe with a similar bleeding risk compared to no anticoagulation. However, the long-term impact of left-sided portal hypertension did not appear to translate into any significant increased risk of GI bleeding. The impact of anticoagulation on iSVT recanalisation rates merits further investigation in large-scale clinical trials.
